# PROMOTING INTRINSIC CAPACITY OF OLDER ADULTS: A SYNERGISTIC APPROACH TO ENHANCE CARE PRESCRIPTION

**DOI:** 10.1093/geroni/igae098.0545

**Published:** 2024-12-31

**Authors:** Yu Zhou, Shuangzhou Chen, Yiting Tang, Feng Chen, Doris Sau Fung Yu

**Affiliations:** The University of Hong Kong, Hong Kong, China (People’s Republic); The University of Hong Kong, Hong Kong, China (People’s Republic); The University of Hong Kong, Hong Kong, China (People’s Republic); The University of Hong Kong, Hong Kong, China (People’s Republic); The University of Hong Kong, Hong Kong, China (People’s Republic)

## Abstract

The United Nations has declared the Decade of Healthy Aging 2021-2030 to prioritize proactive and collaborative strategies for older adults to live well. The World Health Organization’s Integrated Care of Older People (ICOPE) framework aims to guide the assessment of intrinsic capacity (IC) and the environment to promote healthy aging. Based on a research initiative that employs the WHO-ICOPE approach to promote healthy aging, a territory-wide ICOPE-based comprehensive health assessment was conducted in Hong Kong. Those with signs of accelerated aging were referred to a 12-week Jockey Club Pathway to Healthy Aging Program. The program adopts a health-social collaborative approach, clinical critical pathway, empowerment-based behavioral modification, and health-orientated peer support to provide person-centered care to optimize the functional status of older adults. From April 2022 to May 2023, a sample of 3,073 community-dwelling older adults [mean age (SD) = 74.7(7.5)] were recruited from eight geographic districts in Hong Kong and assessed with validated instruments. Latent class analysis identified five IC functional profiles of older adults according to the ICOPE framework. These five patterns include (1) compromised psycho-cognitive functional class, (2) compromised musculo-mobility functional class, (3) compromised metabolic-mobility functional class, (4) compromised nutritional-metabolic equilibrium but preserved functional class, and (5) preserved functional class. After controlling for the demographic profile, chronic disease burden and polypharmacy, the identified IC classification significantly predicted the health-related quality of life and frailty status of the older adults. The study provide new insights on the development of an integrative assessment and care planning for healthy aging promotion.

